# NMR and Electrochemical Investigation of the Transport Properties of Methanol and Water in Nafion and Clay-Nanocomposites Membranes for DMFCs

**DOI:** 10.3390/membranes2020325

**Published:** 2012-06-20

**Authors:** Isabella Nicotera, Kristina Angjeli, Luigi Coppola, Antonino S. Aricò, Vincenzo Baglio

**Affiliations:** 1 Department of Chemistry, University of Calabria, 87036 Rende (CS), Italy; Email: kristina.angjeli@unical.it (K.A.); lg.coppola@unical.it (L.C.); 2 CNR-ITAE Institute, via Salita S. Lucia sopra Contesse, 5, 98126 Messina, Italy; Email: arico@itae.cnr.it (A.S.A.); baglio@itae.cnr.it (V.B.)

**Keywords:** self-diffusion, NMR, DMFC, nanocomposites, clays, transport properties, methanol

## Abstract

Water and methanol transport behavior, solvents adsorption and electrochemical properties of filler-free Nafion and nanocomposites based on two smectite clays, were investigated using impedance spectroscopy, DMFC tests and NMR methods, including spin-lattice relaxation and pulsed-gradient spin-echo (PGSE) diffusion under variable temperature conditions. Synthetic (Laponite) and natural (Swy-2) smectite clays, with different structural and physical parameters, were incorporated into the Nafion for the creation of exfoliated nanocomposites. Transport mechanism of water and methanol appears to be influenced from the dimensions of the dispersed platelike silicate layers as well as from their cation exchange capacity (CEC). The details of the NMR results and the effect of the methanol solution concentration are discussed. Clays particles, and in particular Swy-2, demonstrate to be a potential physical barrier for methanol cross-over, reducing the methanol diffusion with an evident blocking effect yet nevertheless ensuring a high water mobility up to 130 °C and for several hours, proving the exceptional water retention property of these materials and their possible use in the DMFCs applications. Electrochemical behavior is investigated by cell resistance and polarization measurements. From these analyses it is derived that the addition of clay materials to recast Nafion decreases the ohmic losses at high temperatures extending in this way the operating range of a direct methanol fuel cell.

## 1. Introduction

The direct methanol fuel cell (DMFC) is a promising alternative power source [1,2] that combines the merits of direct hydrogen/air fuel cells with the advantages of a liquid fuel, such as convenient handling and high energy density. Despite these advantages, technical barriers still need to be overcome for their large scale commercialization of DMFCs [3]. One of such technical barriers is the methanol crossover, or the direct transport of methanol from anode to cathode via the proton exchange membrane. Methanol crossover will not only lead to reduced energy conversion efficiency because of the direct chemical oxidation of methanol in the cathodic half-cell that hence drastically degrades fuel cell performance but also cause fuel cell operational failures. It is essential to investigate the mechanisms of the methanol transport through the electrolyte in order to gain a better understanding of the crossover mechanism.

In recent years, increasing interest has been devoted to the development of high temperature PEMFC systems. In fact, most of the key issues and shortcomings of the PFSA-based PEMFC technology, such as water management, CO poisoning, cooling and heat recovery, can be solved or avoided by developing alternative membranes with suitable ionic conductivity and stability up to 120–130 °C. Polymer membranes able to operate above 120 °C could benefit from both enhanced carbon monoxide (CO) tolerance and improved heat removal. The most significant barrier to running a polymer electrolyte fuel cell at elevated temperatures is maintaining the proton conductivity of the membrane. Higher temperature increases the water vapour pressure required to keep a given amount of water in the membrane, thereby increasing the likelihood that water loss will occur and significantly reduce proton conductivity. The conductivity of a dry membrane is several orders of magnitude lower than a fully saturated membrane. A number of alternative strategies [4,5,6,7] have been proposed to satisfy these requirements and to maintain membrane conductivity in a dehydrating environment (*i.e.*, elevated temperature and reduced relative humidity), such as composite membranes containing finely dispersed hygroscopic inorganic oxides [8,9,10,11,12] or heteropolyacids [13]. The properties of these composite membranes not only depend on the nature of the ionomer and the solid used but also on the amount, homogeneous dispersion, size, and orientation of the solid particles dispersed in the polymeric matrix. Additionally, the introduction of inorganic materials into the polymeric matrix can be considered as a good approach to improve the proton transport, the retention to the swelling and the resistance to the methanol cross-over in DMFC working conditions (membrane in equilibrium with liquid water-methanol mixture).

Recent studies have shown that polymer nanocomposites based on layered clay nanoparticles exhibit reduced gas permeability due to the presence of impermeable clay platelets as well as structural changes in the polymer induced by the clay nanofillers [14,15,16]. Additionally, our recent study [11] has demonstrated as such smectite clays materials dispersed in Nafion significantly improve the water retention and mobility at temperatures above 100 °C of the nanocomposite membrane.

Clay minerals are among the most important soil constituents. The structure, chemical composition, exchangeable ion type and small crystal size of smectite clays are responsible for several unique properties, including a large chemically active surface area, a high cation exchange capacity, interlamellar surfaces having unusual hydration characteristics, and sometimes the ability to strongly affect the flow behaviour of liquids. These materials demonstrate high chemical and thermal stability; in fact, they are very attractive for numerous industrial applications such as adsorbents, ion exchangers, pharmaceutical additives, fertilizers, *etc.* Concerning the polymer nanocomposites, the first practical application of a nanocomposite was the nylon-montmorillonite clay system that displayed a large increase in tensile strength, modulus, and heat distortion temperature without loss of impact resistance [17]. Since then, the field has rapidly expanded using different polymer/clay systems, giving rise either to exfoliated or to intercalated systems [15,18,19].

Smectite clays are a class of layered aluminosilicate minerals and consist of an octahedral alumina layer fused between two tetrahedral silica layers (about 1 nm) [20,21]. The monovalent ions located between the clay layers allow the absorption of polar solvent, like water, with good retention capacity so, when incorporated into a polymer membrane, they help to prevent the loss of the hydration water not only at high temperatures but also under low relative humidity environment. Moreover, the properties of the smectite nanoclays can be tailored using simple chemical methods such as intercalation with organic or inorganic guest molecules. Their surface properties, for example, can be easily modified through treatment with an organic surfactant. As a result the presence of the surfactant expands the interlayer gallery rendering the nanoclay compatible with hydrophobic media and polymer matrices.

Typical preparation methods of these nanocomposites include solution, melt intercalation, and *in situ* polymerization [22], from which we can obtain conventional, intercalated or exfoliated nanocomposites. We have verified that the solution intercalation method is very effective in incorporating exfoliated clays into Nafion polymer, in which the individual clay layers lose their stacking and are uniformly dispersed in the continuous polymeric matrix. In this study, we want to verify if, as soon as exfoliated nanocomposites are formed, the barrier properties of polymer are increased (due to less surface area and longer pathway) leading not only to low gas (O_2_, air) permeability but, reducing the fuel crossover. In fact, in the development of new low temperature PEMs for use in the DMFC, two of the main concerns are proton-ion and methanol-transport through the membrane. The objective is to significantly reduce the methanol transport whilst having and maintaining high proton transport. The research concerning the polymer electrolytes is aimed to the development hybrid morphology in order to promote the Grotthus [23] jump proton transport mechanism and minimize the Vehicle mechanism [24,25]. 

In this work, one synthetic (Laponite) and one natural clay (SWy-2 montmorillonite) with different structural and physical parameters were chosen as nanofillers: Laponite shows both low layer charge density (CEC = 48 molar equiv/100 g) and particle size (20 nm), SWy-2 has a high layer charge density (CEC = 76.4 molar equiv/100 g) and particle size (200 nm). Composite membranes were synthesized by solution intercalation with 3% of filler loading with respect to the polymer.

We have investigated the transport properties of the water and methanol within the electrolyte membranes both as a function of methanol concentration and as a function of temperature by NMR methods. The aim is to understand the molecular dynamics and the mechanisms that are at the basis for ionic conduction and how these are influenced by polymeric structure and by nanofillers added to the polymer, through direct measurement of the self-diffusion coefficients (D) and relaxation times (T1). The explored temperature range was from 20 °C up to 130 °C and for two methanol-water solution concentrations, 2 M and 4 M.

A preliminary electrochemical characterization was carried out on the filler-free and composite membranes by using ac-impedance spectroscopy and polarization measurements, in order to validate the NMR results.

## 2. Results and Discussion

### 2.1. Uptake and Swelling Properties of the Nafion and Nanocomposites Membranes

The electrolyte membranes were swelled by immersion in pure methanol, pure water, and in aqueous methanol solutions at two different alcoholic concentration, 2 M and 4 M.

The swelling properties of the membranes show marked differences with respect to used solvents, with an increasing of the uptake, consequently of the swelling, as the solution concentration increases, up to reach an uptake of over 150% by mass and a doubling of the membrane size if we use pure methanol. This implies a significant limit to utilizing high concentrations of methanol solutions in the DMFCs. Table 1 shows some data concerning the saturation (maximum) uptake values of the filler-free Nafion and the clay-nanocomposites membranes. It is evident that the effect of the nanofillers is to promote an increase of the membrane water uptake (from 24 wt.% of Nafion up to 45 wt.% of the Swy-composite), but there is also a big effect on the methanol solutions, in fact, membranes can reach an uptake of 60 wt.% when swelled in 4 M methanol solution.

**Table 1:** Methanol solution and water uptake (wt.%) of the membranes at room temperature.

Membranes	Uptake (wt.%) 4M solution	Uptake (wt.%) 2M solution	Uptake (wt.%) pure H_2_O
filler-free Nafion	34	28	24
**Lap**/Nafion	60	46	30
**Swy**/Nafion	60	56	45

### 2.2. ^1^H-NMR Study: D, T_1_ and Spectra Lineshapes

In this study we used NMR methods to investigate the transport properties of water and methanol molecules confined in the porous structure of Nafion and composite membranes and in order to check the effect of the clay-fillers added. 

**Figure 1:**
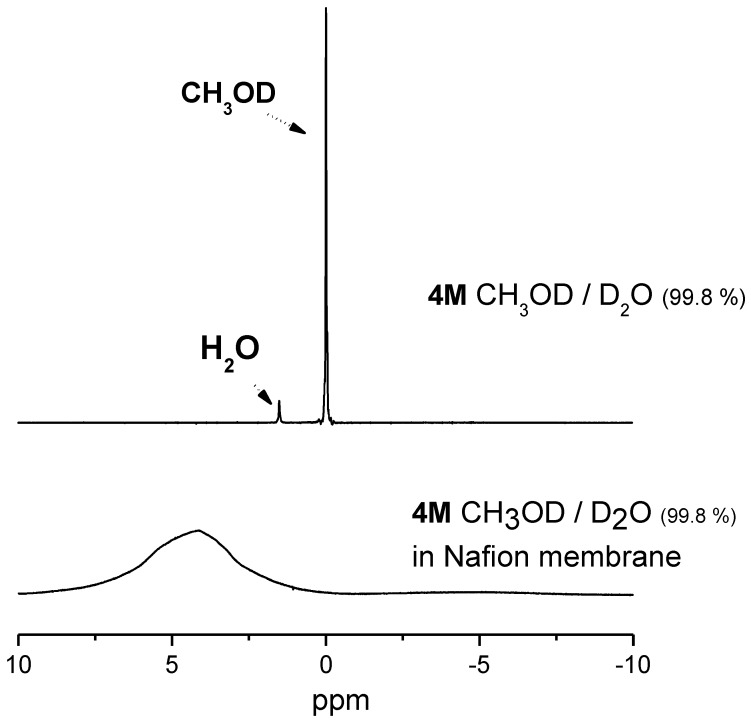
^1^H-NMR spectra recorded on 4 M aqueous methanol solution (CH_3_OD/D_2_O) as prepared and confined in Nafion membrane, respectively. The chemical shift variation of the methanol's signal is caused by the different chemical environment and the strong interactions with the polymer backbone.

It is often possible in methanol–water mixtures to resolve spectroscopically the methyl and hydroxyl protons in an NMR experiment which, in principle, should permit the measurement of both water and methanol diffusion. However, in the present case of solvents confined in membranes, due to the linewidth of the ^1^H-NMR signals, it is not possible to distinguish, through their chemical shift, methanol and water (see Figure 1). 

In order to discriminate between the NMR signals of methanol and water, the membranes were equilibrated in solutions prepared with deuterated molecules, *i.e.*, mixture of CH_3_OD/D_2_O and CD_3_OD/H_2_O. The reason to use CH_3_OD instead of CH_3_OH is due to the fast rate exchange of hydroxyl groups between water and methanol molecules during the “NMR times”, which could affect the measurements. 

Hence, we used the only available signal coming from the methyl groups to perform the NMR diffusometry and relaxometry measurements of methanol confined inside the electrolytes films. With regard to the deuterium signal, this nucleus has a spin-spin relaxation time (T_2_) which is very short, so it is practically impossible to perform measurements of diffusion through the PGSE sequence. For this reason, in order to achieve complete information on the solvents mobility (both water and methanol in mixture), membranes were also swollen in solutions of CD_3_OD-H_2_O, to have only the proton's signal from the water molecules.

In summary, Table 2 reports the solvents and mixtures used to swell the membranes for the NMR study; the solvent marked in bold, is that “seen” by NMR.

**Table 2:** Solvents and solutions used to prepare swollen membranes for the NMR study.

solvents	concentration
**CH_3_OD**	pure
**H_2_O**	pure
**CH_3_OD**/D**_2_**O	2M
CD **_3_**OD/**H_2_O**	2M
**CH_3_OD**/D**_2_**O	4M
CD **_3_**OD/**H_2_O**	4M

**Figure 2:**
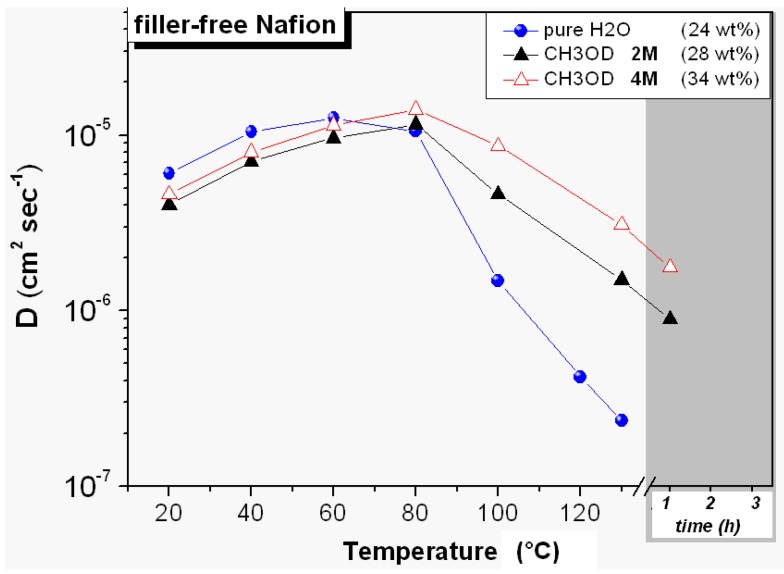
Self-diffusion coefficients of water and methanol (2 M and 4 M solution concentration) confined in filler-free Nafion membranes from 20 °C up to 130 °C; the data collected at 130 °C after 1 h is also plotted. In the legend, the water and solution uptakes are indicated.

Figure 2 displays the diffusion coefficients of water and methanol (in 2 M and 4 M solutions) measured in the swollen membrane of filler-free Nafion, in the range 20–130 °C. Concerning the water diffusion, as expected, at temperatures over 60–80 °C, it decreases rapidly due to the water evaporation from the membrane, which is the main limitation of Nafion in order to work at high temperatures. The methanol diffusion is slightly lower than water up to 60 °C, both at 2 and 4 M concentration but, over this temperature, it remains always higher. For instance, at 130 °C, while the water signal and diffusion are very low, the methanol maintains both NMR signal and diffusion coefficients fairly well, even after 1 h at the same temperature (without any external humidification). We groped to explain this result by considering the distribution of the solvents (water and methanol) inside the ionic pores of Nafion membranes and the transport mechanism involved in the water/methanol diffusion. During the swelling, a certain amount of water is involved in the primary hydration shells of the SO_3_^−^ groups, while most of the additional water fills the volumes of pores forming higher order hydration layers and behaves more bulk-like [26,27] Methanol molecules are, instead, collected adjacent to the polymer backbone with a less favorable complexation of protons as compared to that by water [28,29]. In other words, as reported in other studies [28,30], a micro-phase separation occurs, where water-rich and methanol-rich spatial domains are created, presumably with a characteristic size in the order of the Nafion pore size. In this description, the molecules of methanol interacting with the polymer, and in particular with fluorine atoms on both the backbone and the side-chains, are distributed on the surface of the pores, while the water molecules are at the center acting, in the maximum swelling conditions, as bulk-like water. This scenario is consistent with: (i) the higher methanol diffusion respect to the water at high temperatures; (ii) the increase of the swelling (higher uptake of the 4 M solution) and also of the methanol diffusion coefficient with increasing methanol concentration.

Differently from the membrane of Nafion recast, that is completely soluble in pure methanol, the composite membrane obtained by Swy-nanoclay at 3 wt.% of filler loading with respect to the polymer, shows a greater stiffness, even if it gets a maximum of 200 wt.% of uptake and a swelling which causes at least a doubling of the size of the film. The same membrane in pure water reaches 45% of uptake and low swelling.

**Figure 3:**
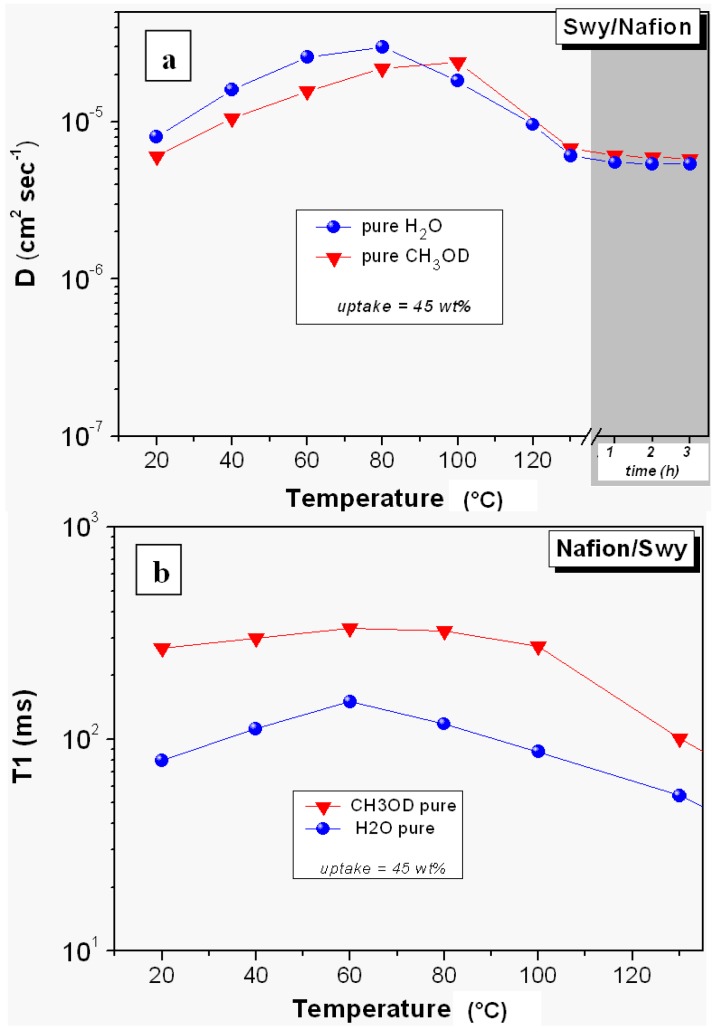
(**a**) Self-diffusion coefficients and (**b**) longitudinal relaxation times (T_1_) of pure water and pure methanol confined in Swy/Nafion nanocomposite membrane from 20 °C up to 130 °C. Data collected at 130 °C after some hours are also plotted in the diffusion graph.

In order to compare the data of diffusion of water and methanol in this membrane, we left to dry the piece of membrane swollen in pure methanol until it reached a weight corresponding to 45 wt.% methanol uptake, and therefore it was put in the NMR tube. Figure 3 displays the diffusion coefficients and the relaxation times (T_1_) of the pure water and pure methanol measured on the swollen Swy/Nafion membrane in the temperature range 20–130 °C.

The first noticeable difference with the recast Nafion is that the water mobility in the Swy/Nafion composite is very high in all temperature ranges investigated. In fact, the diffusion increases linearly up to 80 °C and soon after, due to the evaporation of a certain amount of water, it decreases slightly but remains constant and especially after several hours at 130 °C. Methanol diffusion follows the same behavior (also part of the methanol over the 80–100 °C evaporates from the membrane) even if, at lower temperatures, its diffusion is lower than water. 

NMR longitudinal (or spin-lattice) relaxation times (T_1_), compared to diffusion, reflects more localized motions including both translation and rotation on a time scale comparable to the reciprocal of the NMR angular frequency (~1 ns). As the molecular correlation time τ_c_ depends on temperature, a minimum in T_1_ is often observed when ωτ_c_ ~1, where ω is the NMR frequency [31]. In the temperature range investigated, well above the T_1_ minimum, *i.e.*, in the so-called extreme narrowing limit (ωτ_c_ << 1), higher T_1_ values suggest more facile molecular rotational and translational motion.

If we observe the temperature dependence of the T_1_ of water and methanol in the Swy-nanocomposite membrane (Figure 3b), we find a much different behavior with respect to the diffusion: methanol relaxes slower than water and the T_1_ values are almost constant in the temperature range 20–100 °C. This implies a greater local mobility (more translational and rotational degrees of freedom) of the methanol molecules, probably due to the weaker electrostatic interactions with the polymer matrix respect to the water molecules: methanol forms a less extensive network of hydrogen bonds in Nafion compared to water.

As a result, the lower diffusion of methanol at temperatures below 100 °C (before a significant evaporation), when a large amount of water and methanol is present, may be explained through the blocking effect of the layered nanoclays dispersed in the polymer matrix. In this temperature range, the *Vehicle mechanism* [24,25], where molecules associated with the proton are dragged along, overrides the *Grotthus* mechanism [23], where proton mobility is connected to rotation of the molecules within a constantly changing network of hydrogen bonds.

Above 100 °C, it is reasonable to expect that the evaporation essentially affects the bulk/“free” solvents, so the biggest contribution to the diffusion comes from the solvent molecules strongly interacting with hydrophilic groups and the polymer backbone, for water and methanol, respectively, resulting in a lower D. In such conditions the structural transport mechanism (*Grotthus*) should be predominant for both solvents, therefore the effect of obstruction by the nanoparticles is less effective. The result is that, despite the local motions of molecules of methanol are faster (higher T1), since it fails to form an extensive network of hydrogen bonds, the diffusion is practically equal to that of water.

From these results, certainly the use of pure methanol is not a practicable choice in the DMFCs because the solvent cross-over is not sufficiently reduced, and there is excessive swelling of the membrane. However, it is important to underline the different water retention behavior of the filler-free Nafion and this composite membrane at high temperatures which suggests that a significant proton conductivity can be ensured by electrolytic membranes in high temperatures and low humidity conditions. Therefore, it would be of great interest to investigate the water and methanol diffusion changing the boundary condition from pure methanol to a methanol/water mixture.

**Figure 4:**
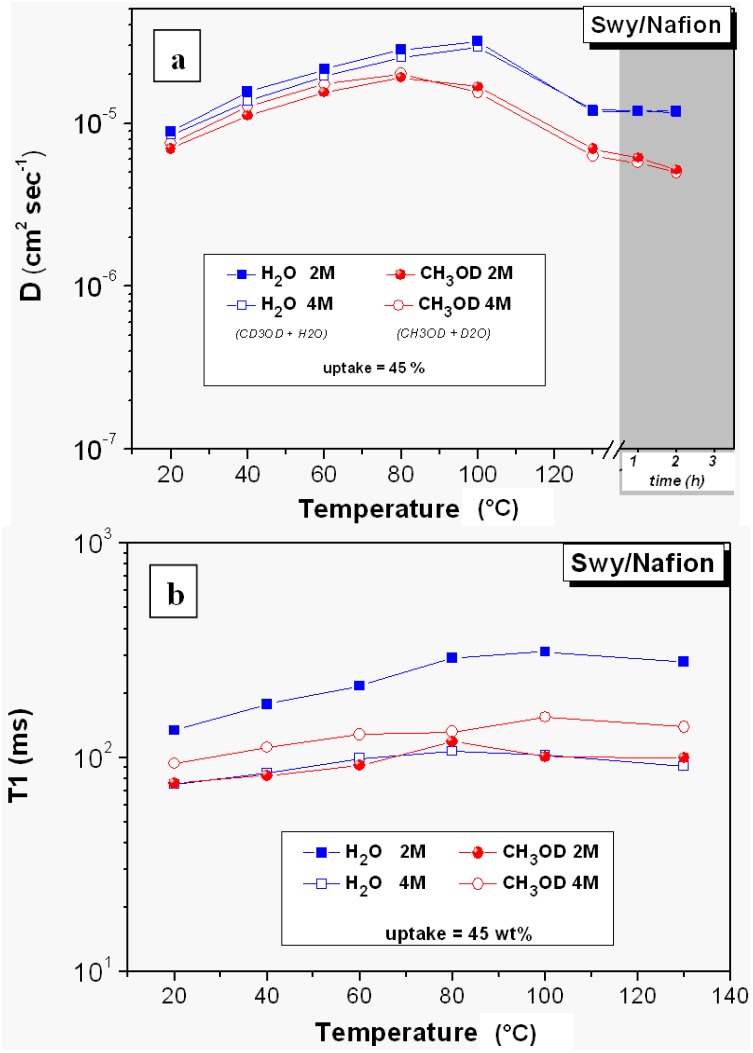
(**a**) Self-diffusion coefficients and (**b**) longitudinal relaxation times (T1) of water and methanol in 2 M and 4 M solutions confined in Swy/Nafion nanocomposite membrane from 20 °C up to 130 °C. Data collected at 130 °C after some hours are also plotted in the diffusion graph.

Figure 4 shows the diffusion and relaxation times of water and methanol in 2 and 4 M solutions, measured in swollen Swy/Nafion nanocomposite membrane, from 20 °C up to 130 °C.

As mentioned previously, two mixtures of deuterated solvents were used: CH_3_OD + D_2_O and CD_3_OD + H_2_O, to perform the NMR experiments on methanol and water, respectively. In such a way we can compare the mobility of water and methanol confined in membrane at the same solution concentration and, for the same reason, we used the same solution uptake value, 45 wt.%.

Water diffusion is always higher than methanol in the whole investigated temperature range and for both solution concentrations. Up to 100 °C the diffusion coefficients of water in 2 M solution are slightly higher than 4 M, accordingly to the water surplus available in the first solution which increases the bulk-like water fraction. When most of this “free” water evaporates, the diffusion coefficients of the “bounded” water (hydrated water) are similar for both concentrations. 

Concerning the methanol diffusion at 4 M concentration, initially it is very close to that of water but, with the increasing of the temperature, it becomes gradually smaller and moves far from water with a significant fall after 80 °C. This abrupt drop is still related to the blocking effect, that becomes a significant factor just when the solution content is low (due to evaporation), *i.e.*, when the hydrophilic domains sizes are reduced and therefore, big particles can obstruct the methanol molecules pathways.

The comparison with the methanol diffusion in 2 M solution describes a similar trend, however already starting from the low temperatures, the diffusion is much lower than water at the same concentration. This is consistent with the vehicular transport mechanism and with the molecular interactions of the methanol molecules with the polymeric sites, the first one increases the water mobility, the second effect reduces the methanol diffusion because molecules are “complexed” on the clusters surface.

The molecular complexation is also visible from the relaxation times (Figure 4b): methanol T_1_ in 2 M solution are lower than 4 M, *i.e.*, the local motions are more impeded and inhibited in the first solution.

**Figure 5:**
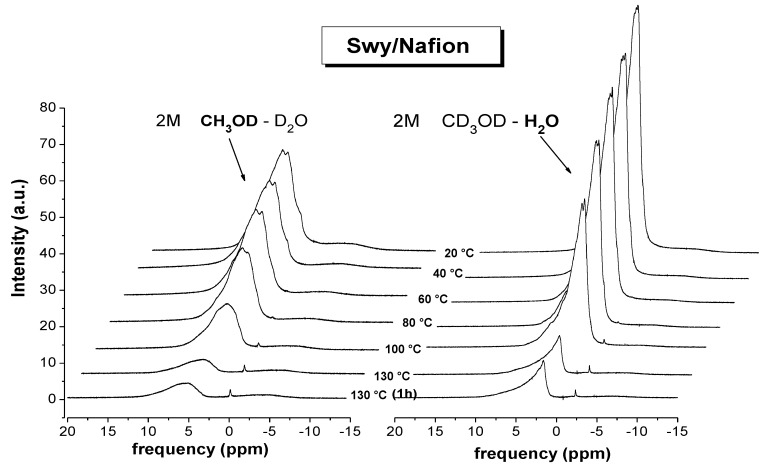
Evolution of proton spectra from methanol (on the left) and water (on the right) as a function of temperature on Swy/Nafion composite swollen in 2 M methanol solution. The spectra were referenced setting methyl protons and pure water at 0 ppm, respectively.

The analysis of the ^1^H-NMR spectra of methanol and water with the temperature increasing can give further information on the solvents distribution in the membrane (hydration and interactions) as well as on the evaporation dynamics. Figure 5 shows the temperature evolution of the proton spectra acquired on Swy/Nafion swollen in 2 M methanol solution, from 20 °C up to 130 °C (spectrum recorded at 130 °C after 1 h is also reported). In the figure, we compare the spectral lines of methanol (on the left) and water (on the right). Both signals are large and asymmetric, typical of a multiple components configuration, however, the FWHM (full width at half maximum) of the water and methanol spectra are quite different: 675 Hz against 1,170 Hz at 20 °C, *i.e.*, the line width of methanol is almost double of the water. 

This implies that, while for the water there is a strong component of bulk which contributes to the “shrinkage” of the spectral line, the molecules of methanol instead are strongly interacting with the polymer (the “complexation” referred to above) promoting a broadening of the line, as well as reducing their mobility on both short and long range (T_1_ and diffusion).

By heating, the intensity of these peaks decreases because of the solvents evaporation from the membrane, with a pronounced drop above 100 °C; at 130 °C the intensity of the residual signal remains constant for several hours (obviously without any supplying humidity), and this is responsible for both the proton and methanol diffusion that we are able to detect at these high temperatures.

The discussion of the data just analyzed is complex but definitely indicates that the microstructure plays a key role in the mass transport of water and methanol.

The microstructure is influenced by the chemical-physical properties of the smectite clays such as the nanoparticles size and the layer charge density. In fact, higher platelets dimensions may promote stronger alterations of the network structure as well as create an obstruction effect, while a high value of surface charge density may facilitate or promote the proton transport through hopping mechanisms.

These assumptions are corroborated by the experimental results obtained on the hybrid membrane based on Laponite nanoclay. This synthetic clay is completely exfoliated in the Nafion polymer but has both smaller CEC and particle size with respect to Swy. 

**Figure 6:**
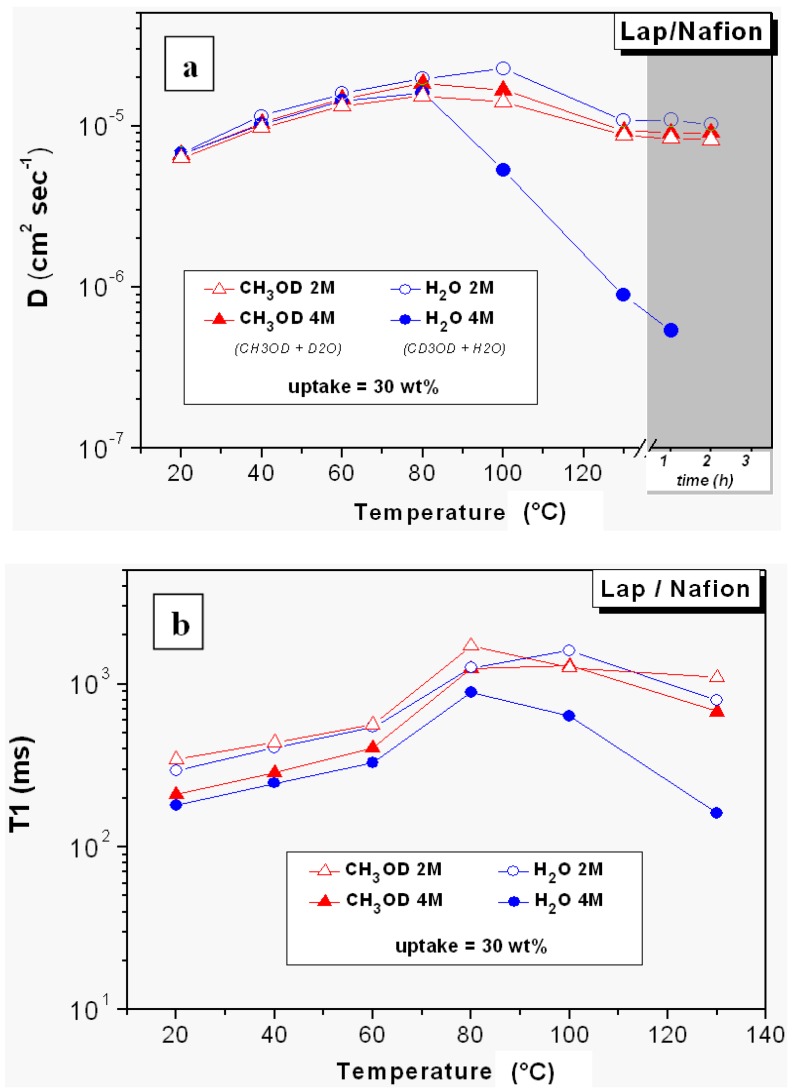
(**a**) Self-diffusion coefficients and (**b**) longitudinal relaxation times (T1) of water and methanol in 2 M and 4 M solutions confined in lap/Nafion nanocomposite membrane from 20 °C up to 130 °C. Data collected at 130 °C after some hours are also plotted in the diffusion graph.

In Figure 6 diffusion coefficients and relaxation times of water and methanol in 2 M and 4 M solutions in the swollen Lap/Nafion nanocomposite membrane (uptake 30 wt.%), from 20 °C up to 130 °C, are reported. Basically, the mobility of the methanol is not influenced by the solution concentration and is very similar to that of water and suggests that no relevant obstruction effect from Laponite nanoparticles acts on the methanol transport.

**Figure 7:**
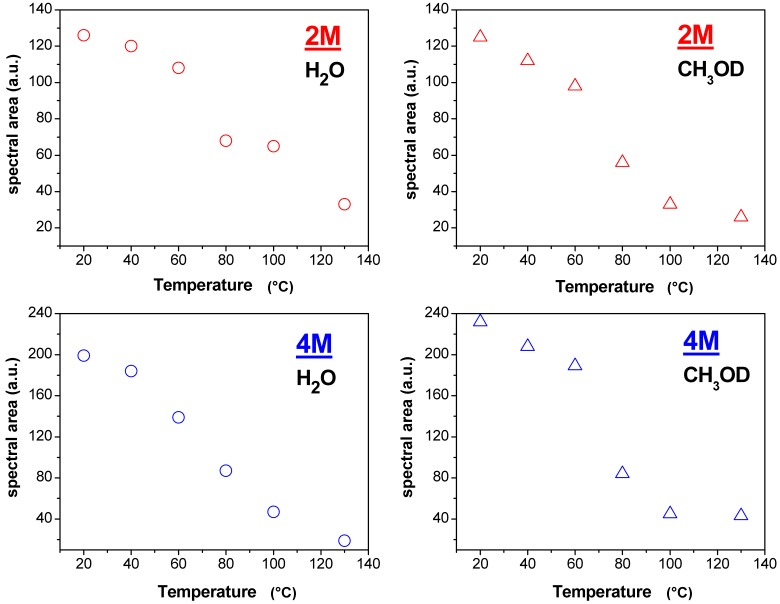
Plots of the spectral area *vs.* temperature of water and methanol in both 2 M and 4M solutions confined in Lap/Nafion nanocomposite.

In order to achieve a complete framework on this membrane and analyze the various differences, we show in Figure 7 the plots of the spectral area *vs.* temperature, of water and methanol in both 2 M and 4 M solutions confined in Lap/Nafion nanocomposite. An evident evaporation of both water and methanol after 60 °C is evident in these graphs producing a remarkable reduction of the NMR signals. The water at 4 M concentration shows a decrease of the peak area which is slightly different because, rather than a net head between 60 °C and 80 °C, there is a clear and constant reduction of the intensity of the proton signal starting at 40 °C.

The behavior of Lap/Nafion membrane is quite different with respect to Swy/Nafion and a comparison of all NMR data sheds light on the following main differences:

(1) The relaxation times of Lap-composite are higher over the whole temperature range, which implies higher local motions (rotations and translations) and therefore, weaker interactions with the filler;
(2) significant evaporation on Lap-composite at 80 °C, evidenced by the strong reduction of the spectra integrals; in Swy-composite, this drop is found at 130 °C;
(3) in Lap-composite, water for the 4 M solution shows a decrease of both diffusion and T1 after 80 °C, while for the 2 M solution, water and methanol diffusion is similar.

The origin of these three outcomes can be correlated to the lower cationic exchange capacity (CEC) of Laponite clay compared to Swy montmorillonite. Fewer hydrophilic “coordinating” sites produce the lowering of the retention capacity of the membrane and, consequently, the water diffusion is reduced. In particular, when we start with a lower amount of water (*i.e.*, 4 M solution), what remains at high temperature is strongly linked water. However, further investigations are necessary to clarify this issue.

### 2.3. Electrochemical Investigation

**Figure 8:**
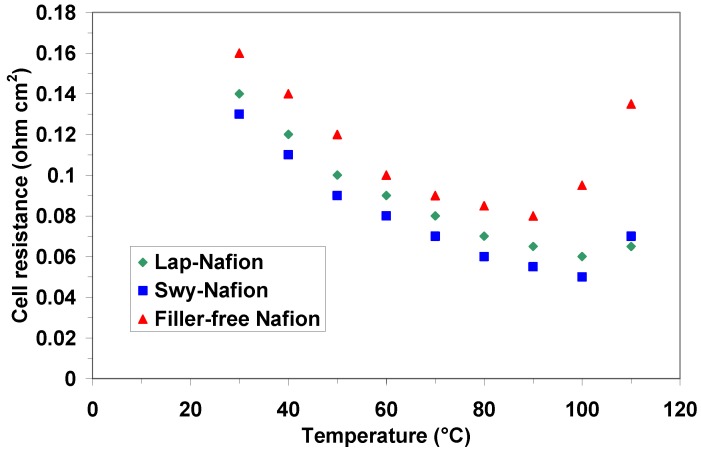
Cell resistance values as a function of temperature for the cells equipped with the different membranes.

The electrochemical investigation was carried out in a single cell by recording the ac-impedance spectra at different temperatures (from 30 °C to 110 °C) and the polarization curves in the temperature range from 90 °C to 110 °C, feeding a 2 M methanol solution at the anode and oxygen at the cathode. The series resistance (high frequency intercept on the real axis in the Nyquist plot) was plotted as a function of temperature as shown in Figure 8. Obviously, the fuel cell conditions are completely different from that of the NMR experiments. However, there are some analogies with the NMR results; in fact, the decrease of cell resistance in the composite membranes indicates an increase of water retention caused by the fillers, in particular for the Swy-based membrane. The introduction of these clays thus produces an extension of the operating temperature of a direct methanol fuel cell. 

**Figure 9:**
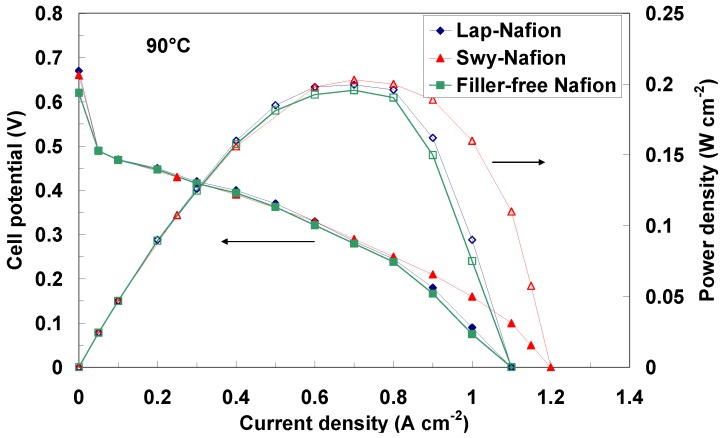
DMFC polarization and power density curves at 90 °C for various membrane-electrode assemblies equipped with composite and filler-free membranes.

**Figure 10:**
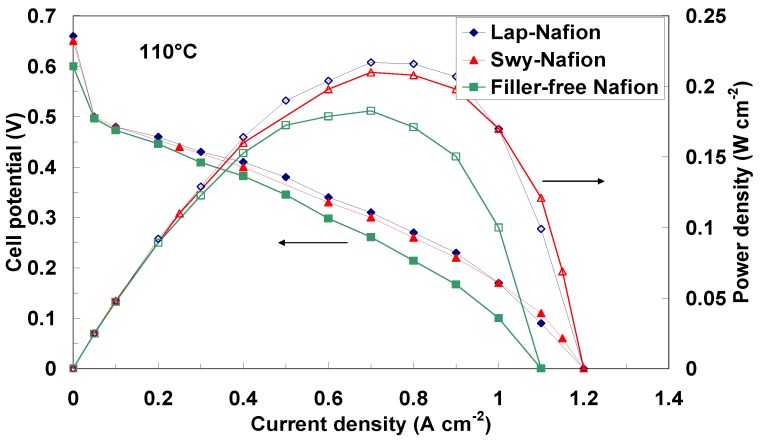
DMFC polarization and power density curves at 110 °C for various membrane-electrode assemblies equipped with composite and filler-free membranes.

In Figure 9 and Figure 10 the polarization and power density curves obtained with the different membranes at 90 °C and 110 °C, respectively, are reported. As can be observed, the maximum power density of the DMFC equipped with the composite membranes increases as the temperature increases, although the methanol solution is fed under atmospheric pressure and thus subjected to evaporation. Unfortunately, these operating conditions (atmospheric pressure) did not reach higher temperatures than 110 °C. However, from this analysis the effect of the water retention properties of the fillers is clear. In fact, the filler-free Nafion-based cell showed a drop of performance with the temperature due to water evaporation and the consequent increase of cell resistance (see Figure 8).

The performance at 90 °C (Figure 9) is very similar for all the membranes and superior compared to previously investigated composite membranes at this temperature [32,33,34,35,36].

However, we have to take into account that different catalysts were used in the present analysis; also the thickness of the membranes was different compared to the composite membranes previously developed by our group [32,33,34,35,36] using various inorganic oxides as fillers. Increasing the temperature up to 110 °C (Figure 10), under atmospheric pressure at the anode, water evaporation produces an increase of cell resistance (see Figure 8) with a consequent decrease of the performance for the filler-free Nafion membrane. Whereas, a slight increase of power density is recorded with the clays-nanocomposites under these conditions, confirming the higher water affinity of these membranes as shown by NMR experiments. 

The remarkable behavior of smectite clays-based nanocomposites at high temperatures is reported in our two recent studies [11,12] where the exceptional water retention and mobility property of composites systems with respect to the recast Nafion is proven. This suggests that a significant proton conductivity can be ensured by electrolytic membranes in high temperatures and, therefore, highly promising also in PEMFCs applications.

Finally, Table 3 shows the variation of the open circuit potential (OCP) as a function of temperature for the various membranes. The OCP is an important parameter in a DMFC in order to assess the methanol permeability of a membrane. The DMFCs based on Lap/Nafion and Swy/Nafion membranes showed similar behavior in the temperature range from 90 °C to 110 °C, whereas the cell equipped with the filler-free membrane evidenced a lower value of OCP. This is a clear indication of the blocking effect of the composite membranes towards methanol cross-over, as already evidenced from NMR results. 

**Table 3:** Open circuit potential (OCP) values at the different temperatures for the various membranes.

Membranes	Open Circuit Potential (V) @ 90 °C	Open Circuit Potential (V) @ 100 °C	Open Circuit Potential (V) @ 110 °C
filler-free Nafion	0.62	0.61	0.60
**Lap**/Nafion	0.67	0.66	0.66
**Swy**/Nafion	0.66	0.66	0.65

As a result, such composite membranes, capable of extending the operating temperature of Nafion membranes, would be a potential solution to some of the drawbacks presently affecting direct methanol fuel cells (DMFCs) as well as reformate-fuelled polymer electrolytes (PEMFCs). Fuel cell operation at elevated temperatures will limit the effects of electrode poisoning by adsorbed CO molecules, increase both methanol oxidation and oxygen reduction kinetics and simplify water and thermal management. Recently, composite Nafion membranes have been also proposed for PEM electrolysers with good results [37,38], demonstrating the high potentiality of these composite materials and the need to understand the water transport mechanism inside them for a further improvement of their properties. 

## 3. Experimental Section

### 3.1. Materials

Two smectites clays with different structural and physical parameters (structural formula, particle size and cation exchange capacity) were used. The first was a natural Wyoming montmorillonite (SWy-2) obtained from the Source Clay Minerals Repository, University of Missouri, Columbia, with a cation exchange capacity (CEC) measured by the Co(II) procedure equal to 80 mequiv per 100 g clay and particle size around 200 nm. The second clay was a synthetic trioctahedral hectorite, Laponite (Lap), produced by Laporte Industries Ltd with the lowest CEC 48 mequiv per 100 g clay and the smallest particle size (20 nm). SWy-2 montmorillonite was fractioned to < 2 μm by gravity sedimentation and purified by well-established procedures in clay science [39]. Sodium-exchanged samples (Na^+^-SWy-2) were prepared by immersing the clay into 1 M solution of sodium chloride. Cation exchange was complete by washing and centrifuging four times with dilute aqueous NaCl. The samples were finally washed with distilled deionized water and transferred into dialysis tubes in order to obtain chloride-free clays and then dried at room temperature.

Finally, Nafion solution (20 wt.% in mixture of lower aliphatic alcohols and water) were purchased from Aldrich.

### 3.2. Composites Membranes Preparation

The composite membranes were prepared from 20 wt.% Naﬁon^®^ solution according to the following processes: (i) 1 g of Nafion solution was heated at about 60 °C to remove all the solvents (water, isopropanol, *etc.*); (ii) Naﬁon resin was redissolved with 10 mL DMF until become a clear solution; (iii) the filler was dispersed in the same solvent under stirring for 1 day and added slowly to the solution of Nafion; (iv) the final solution was ultra-sonicated for 1 h and then stirred at 60 °C to ensure complete mixing; finally (v) casting on a petridisk at 80 °C overnight was performed in order to obtain a homogeneous membrane. The hybrid membrane is removed from the petridisk by immersing the glass plate in deionised water for several minutes. In order to reinforce the membrane, this was sandwiched and pressed between two Teflon plates and put in oven at 150 °C for about 15 min. Composite membranes produced by casting were subsequently treated by rinsing in: (i) boiling HNO_3_ solution (1 M) for 1 h to oxidize the organic impurities, (ii) boiling H_2_O_2_ (3 vol.%) for 1 h in order to remove all the organic impurities, (iii) boiling deionized H_2_O for 40 min three times, (iv) boiling H_2_SO_4_ (0.5 M) for 1 h to remove any metallic impurities, and again (v) boiling deionized H_2_O for 40 min twice to remove excess acid. According to McMillat *et al.*, [40] an ulterior purification procedure was performed in order to ensure the removal of paramagnetic contaminants which are particularly damaging to an NMR experiment, such as the presence of copper that we found by Electron Paramagnetic Resonance analysis. By this procedure membranes were soaked in EDTA solution (0.001 M) for 1 day after followed by a thorough rinse. Then soaked in 2 M HCl at a temperature of 80 °C for 2 h followed by boiling in fresh distilled-deionized water to remove any residual acids and again repeated the treatment with EDTA. Finally, rinsing in boiled deionized water three times to remove residual EDTA and stored at room temperature at fully hydrated state.

### 3.3. Characterization Techniques

NMR measurements were performed using a Bruker AVANCE 300 wide bore NMR spectrometer working at 300 MHz on 1H and the spectra were obtained by transforming the resulting free-induction decay (FID) of single π/2 pulse sequences. Self-diffusion coefﬁcients of water were performed with a Diff30 NMR probe by using the pulsed gradient spin-echo (PGSE) method [41]. This technique consists of two rf pulses, Hahn-echo sequence *π/2* *− τ* *− π* with two identical pulsed-gradients, the first applied between the 90° and 180° rf pulse (during the dephasing) and the second after the 180° rf pulse (during the rephasing) but before the echo. Following the usual notation, the gradients have magnitude *g*, duration *δ*, time delay ∆, and the factor (∆ − (*δ*/3)) is the diffusion time over which the molecular displacements are monitored. The attenuation of the echo amplitude is represented by the Stejskal-Tanner equation:

*A* = exp[−*γ*^2^*g*^2^*Dδ*^2^(∆ − (*δ*/3)]

where *D* is the self-diffusion. For the investigated samples, the experimental parameters, ∆ and *δ*, are 10 and 1 ms, respectively. The gradient amplitude, *g*, varied from 10 G·cm^−1 ^to 600 G·cm^−1^. All PGSE data were well described by a single exponential and, for accurate treatment of experimental error, D was determined by regressing the theoretical echo-attenuation onto the experimental data (*i.e.*, resonance integrals). Finally, longitudinal relaxation times (T_1_) of water were measured on the same spectrometer by the inversion-recovery sequence (*π– τ* *− π*/2). Both self-diffusion and T_1_ measurements were conducted by increasing temperature step by step from 20 °C to 140 °C, with steps of 10 °C, and leaving the sample to equilibrate for about 20 min. 

Prior to the NMR measurements, membranes were dried in oven, weighed and then immersed in the various aqueous methanol solutions, in pure water and in pure methanol, at room temperature (in the results section it will be explicated the different kind of solutions prepared and the motivations). Upon being removed from the solutions they were quickly blotted dry with a paper tissue (to eliminate most of the free surface liquid). The liquid content value was determined using a microbalance and recorded as: *uptake% = [(m_wet_* *− m_dry_)/m_dry_]* *× 100.*

At this point the membranes were loaded into a 5 mm NMR Pyrex tube and sealed. In order to minimize the evaporation of the solvent from the membrane with increased temperature, a “cap” of Teflon was placed just above the membrane. Thus, the free volume available to the evaporated solvent was minimized.

For the electrochemical characterization, the electrodes used were composed of commercial gas-diffusion layer-coated carbon cloth for high temperature (HT-ELAT, E-TEK) and low temperature operation (LT-ELAT, E-TEK) at the anode and cathode, respectively. Unsupported Pt-Ru (Johnson-Matthey) and Pt (Johnson-Matthey) catalysts were mixed under ultrasounds with 15 wt.% Nafion ionomer (Ion Power, 5 wt.% solution) and deposited by a doctor blade technique onto the diffusion media for the anode and cathode, respectively. A Pt loading of 2 mg·cm^−^^2^ was used for all MEAs, both at the anode and cathode. The MEAs were obtained by a hot pressing method between electrodes and the different membranes (filler-free recast Nafion, Lap-Nafion and SWy-Nafion composite membranes) at 130 °C and 30 kg·cm^−^^2^ for 1.5 min. The thickness of the membranes used for the electrochemical tests was about 50 µm. The MEAs were tested in a 5 cm^2^ single cell (Fuel Cell Tech., Inc.) connected with an Autolab PGSTAT 302 Potentiostat/Galvanostat (Metrohm) equipped with a frequency response analyzer (FRA) module of impedance. The impedance measurements were performed under potentiostatic control in a frequency range between 20 kHz and 0.1 Hz by frequency sweeping in the single sine mode. The amplitude of the sinusoidal excitation signal was 0.01 V. The series resistance was determined from the high frequency intercept on the real axis in the Nyquist plot. A 2 M methanol solution was fed at the anode under atmospheric pressure with a flow rate of 3 mL·min^−^^1^, whereas oxygen was fed at the cathode at a flow rate of 100 mL·min^−^^1^ under 1.5 bar of pressure. The performance of each MEA was measured under steady-state conditions in the temperature range 90 °C–110 °C.

## 4. Conclusions

PGSE and relaxometry in NMR are powerful methods that allow for the measurement of multicomponent diffusion and mobility on a molecular scale. Using this technique, self-diffusion coefficients and spin-lattice relaxation times of methanol and water in Nafion and nanocomposite membranes were measured as a function of methanol solution concentration and temperature. In order to discriminate between the NMR signals of methanol and water, the membranes were equilibrated in solutions CH_3_OD/D_2_O and CD_3_OD/H_2_O, at 2 M and 4 M concentrations. 

The results achieved on the filler-free Nafion and on two hybrid membranes based on smetic clays, Laponite and Swy-2, with different particle size and cation exchange capacity (CEC), have been analyzed and compared.

In the filler-free Nafion, the methanol diffusion, both at 2 M and 4 M concentration, is higher than water and, at high temperature (100–130 °C), when the water signal almost disappears, the methanol still maintains a discrete mobility. 

Swy montmorillonite particles inside the membrane demonstrate being a physical barrier for methanol cross-over, reducing the methanol diffusion with an evident blocking effect yet, nevertheless ensuring a high water mobility up to 130 °C and over several hours. However, the solution uptake data highlight a strong swelling membrane effect demonstrating the high chemical affinity of the methanol towards the polymer that allows a diffusion, even if lower than water, up to temperatures above 100 °C and that increases with increasing methanol solution concentrations.

Nanocomposite membrane based on the Laponite clay showed a different behavior both for water and methanol with respect to Swy. The smaller CEC and particle size of this synthetic clay result in both lower water retention capacity of the membrane and obstruction toward the methanol, respectively. This is confirmed by the slightly higher cell resistance of the cell based on this membrane compared to Swy-based membrane by using 2 M methanol concentration. 

We conclude that, despite the obstruction effect due to the dispersion of suitable hydrophilic layered nanoparticles in Nafion, the main contribution to the diffusion of methanol is its high uptake in the polymer. Therefore, to enhance the performance and the efficiency of a DMFC, it is necessary to develop membranes which absorb less methanol while maintaining, however, a high proton conductivity.
