# Erratum: Nicotera, I.; Angjeli, K.; Coppola, L.; Aricò, A.S.; Baglio, V. NMR and Electrochemical Investigation of the Transport Properties of Methanol and Water in Nafion and Clay-Nanocomposites Membranes for DMFCs. *Membranes* 2012, *2*, 325–345

**DOI:** 10.3390/membranes6020032

**Published:** 2016-06-11

**Authors:** Membranes Editorial Office

**Affiliations:** Klybeckstrasse 64, CH-4057 Basel, Switzerland

It has been brought to our attention that Laponite^®^ is a trademark of BYK Additives, however the trademark symbol is missing in [1]. We apologize for an inconvenience caused by this omission. The symbol will be added to the online version of the article.
